# Full-Scale Microfiltration for Drinking Water: A Long-Term Performance Analysis

**DOI:** 10.3390/membranes16060212

**Published:** 2026-06-20

**Authors:** Małgorzata Kabsch-Korbutowicz, Małgorzata Wolska, Anna Solipiwko-Pieścik

**Affiliations:** Faculty of Environmental Engineering, Wroclaw University of Science and Technology, Wybrzeże Wyspiańskiego 27, 50-370 Wrocław, Poland; malgorzata.wolska@pwr.edu.pl (M.W.); anna.solipiwko-piescik@pwr.edu.pl (A.S.-P.)

**Keywords:** polymeric membrane, membrane aging, permeability decrease, microfiltration

## Abstract

Microfiltration membranes are widely used in drinking water treatment due to their high efficiency. However, long-term operation of polymeric membranes may lead to deterioration of hydraulic properties as a result of fouling and material aging. This study aims to determine the impact of long-term aging on hydraulic permeability and separation properties, and to determine the lifespan of microfiltration polyvinylidene fluoride (PVDF) membranes. The practical and industrial novelty of this study lies in providing an authentic, 11-year operational baseline for a full-scale microfiltration system treating highly variable surface water. The study evaluates membranes installed in a full-scale plant in Jarosław (Poland), treating surface water from the San River. The system includes 120 PVDF capillary modules (0.1 μm). After 11 years, the membranes maintained very high separation efficiency, ensuring almost complete removal of turbidity and microorganisms. However, membrane resistance increased nearly threefold, while permeability decreased by about 86%. Maintaining capacity required a gradual increase in transmembrane pressure. The permeability loss exceeded the commonly accepted replacement threshold of 70%, suggesting that membrane replacement after more than a decade of operation is technically and economically justified.

## 1. Introduction

Ensuring stable access to safe drinking water constitutes one of the critical challenges of environmental engineering in the 21st century. Faced with the progressive degradation of surface water quality and increasingly stringent regulations regarding water intended for human consumption, conventional treatment methods—typically based on coagulation, sedimentation, and sand filtration—are often proving insufficient. A particular challenge remains the effective removal of fine suspended solids, colloids, and chlorine-resistant pathogens, such as Giardia cysts or Cryptosporidium oocysts.

In response to these limitations, the last few decades have seen rapid development in low-pressure membrane technologies. Microfiltration (MF) and ultrafiltration (UF) have achieved the status of key technologies in modern water treatment plants (WTPs), offering a reliable physical barrier against particulate and microbiological contaminants. The primary advantage of MF or UF systems is their ability to produce permeate of high and consistent quality, largely independent of periodic fluctuations in raw water quality, which is particularly crucial for surface water intakes. Microfiltration membranes have been used in drinking water production for approximately 40 years, with large-scale municipal use beginning in the early 1990s. The impetus for introducing microfiltration into the water treatment system was a massive *Cryptosporidium* outbreak in Milwaukee in 1993 (which affected over 400,000 people) [1]. Because microfiltration is highly effective at removing such parasites, the industry pivoted toward it. The first large-scale municipal MF plant (19,000 m^3^/d) opened in San Jose, California, in 1994 [2]. Since then, microfiltration has been increasingly used for both water and wastewater treatment. It is estimated that 42–45% of the cumulative capacity of installed microfiltration membranes is dedicated specifically to water treatment and wastewater purification, with the remainder divided between biopharmaceuticals and food/beverage processing [3]. The growing popularity of separation using microfiltration membranes for water purification is, among other things, attributed to their usefulness in removing microorganisms from water, which results in a 25% reduction in the required doses of chemical disinfectants [4]. The global market for membrane filtration modules is projected to grow from USD 11.8 billion in 2025 to USD 23 billion by 2034, with MF typically accounting for approximately 20–30% of the total value of the global membrane filtration market [5]. In addition to the advantages of the low-pressure membrane processes associated with the higher quality of treated water compared to conventional surface water treatment techniques, attention should also be paid to economic and environmental aspects. As shown by Hussein et al. [6], a membrane-based WTP uses 48.6% less land than a conventional process-based plant. The capital expenditure (CAPEX) and operating expenditure (OPEX) of a membrane WTP, calculated per cubic meter of treated water, are approximately 5.7% and 11% higher, respectively, than those of a conventional system; however, energy consumption in a membrane plant is about 19% lower. Although the water recovery rate in a classical treatment train is slightly higher than in a membrane-based system (93.4% vs. 92%), the waste stream from the membrane system is less concentrated and does not contain reagents (e.g., harmful Al salts) added during water treatment in the conventional system. Comparable economic indicators were estimated by Makowska and Krauze [7].

Despite their undeniable process advantages, the full-scale operation of polymeric membranes is associated with significant challenges, most notably membrane fouling and material aging. While fouling can be periodically controlled through backwashing and chemical cleaning (Clean-in-Place, CIP), long-term exposure to chemicals (acids, bases and oxidants) and cyclic mechanical stresses lead to irreversible changes in the polymer structure [8,9]. This process results in a permanent decline in performance, a reduction in mechanical strength, and, ultimately, the need for costly module replacement. According to Robinson and Bérubé [10], membrane resistance and fouling rate showed no noticeable changes during the first 5 years of operation. Beyond this period, however, both parameters increased markedly. A similar effect was observed by Yu et al. [11] after seven years of membrane operation. In comparison, Touffet et al. [12] found that membrane resistance declined as the membrane aged, whereas the fouling rate increased over time. Chang et al. [13] have found that among 106 full-scale drinking water membrane plants, membrane service life typically ranged from 5 to more than 10 years. About 37% of the facilities replaced their membranes after roughly 7 years of operation, whereas 13% reported membrane replacement only after exceeding 10 years of use. To extend membrane service life and thereby reduce operational costs, it is essential to understand the factors influencing their degradation and to realistically assess their potential lifespan. Attempts are also being undertaken to chemically regenerate polymer membranes after many years of use [14], which would extend their service life and contribute to a reduction in costs of approximately 3.0 $/m^2^-membrane/year and 0.059 kg CO_2_-e/membrane module CO_2_ emissions, compared to the most commonly used procedures, i.e., replacing membranes with new ones.

In Poland, the application of low-pressure membrane filtration has transitioned from pilot-scale studies to full-scale implementation. Notable examples include the water treatment plants in Sucha Beskidzka (130 m^3^/h), Jarosław (470 m^3^/h), and Andrychów (250 m^3^/h), which have successfully operated membrane systems for over a decade, treating variable-quality surface waters [15,16,17].

Although membrane degradation phenomena have been widely described in the literature, most studies are limited to laboratory-scale experiments or short-term pilot tests [18,19]. There are very few [10,11,13] comprehensive analyses of the behaviour of low-pressure polymer membranes in real industrial conditions over several years of operation.

This paper presents the results of an assessment of microfiltration membranes after 11 years of continuous operation at a surface water treatment plant in Jarosław (Poland). A detailed comparison of the separation and transport properties of membranes was carried out for the periods 2014–2016 and 2021–2025. These periods encompassed the early stage of membrane operation and the period immediately before membrane replacement, allowing for the evaluation of long-term changes in membrane properties under full-scale operating conditions.

The practical and industrial novelty of this study lies in providing an authentic, 11-year operational baseline for a full-scale microfiltration system treating highly variable surface water. The aim of this study is to determine the impact of long-term aging on hydraulic permeability and separation properties and to determine the lifespan of microfiltration PVDF membranes commonly used in drinking water treatment.

## 2. Materials and Methods

### 2.1. Full-Scale DWTP Using MF Membranes

The water treatment plant in Jarosław (southeastern Poland) collects surface water from the San River and treats it using the following processes: batch coagulation, sedimentation, rapid sand filtration, microfiltration (MF), and final chemical disinfection. For coagulation, polyaluminium chloride PAX18 (Kemipol, Wrocław, Poland) is used. During the study period, a variable coagulant dose ranging from 7.2 to 9.4 g Al/m^3^ was applied. The coagulant dosage was adjusted according to the raw water quality.

**Figure 1:**
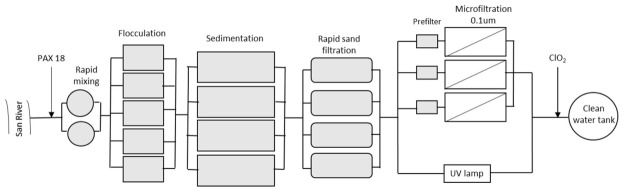
Water treatment train in Jarosław WTP.

The system is also equipped with a UV disinfection system. The nominal capacity of the plant is 470 m^3^/h. The scheme of the technological system is shown in Figure 1.

*Membranes*

**Figure 2:**
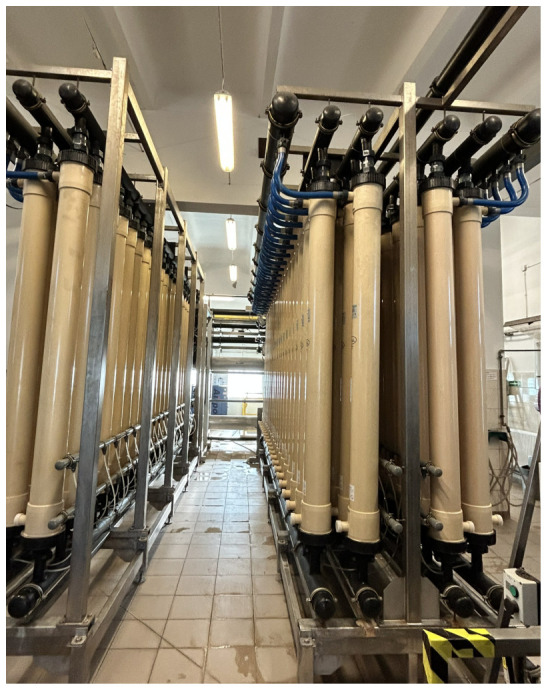
MF system in Jarosław WTP.

Microfiltration water treatment at the WTP in Jarosław is carried out using microfiltration polymer capillary membranes with a pore diameter of 0.1 µm. The installation is equipped with 120 MICROZA UNA-620C (WTR-620C) modules manufactured by Pall Corporation (Port Washington, NY, USA) (currently Asahi Kasei Corporation (Tokyo, Japan)), arranged in 3 blocks/sets of 40 modules each (Rack1, Rack2, and Rack3): Rack1 is equipped with Japanese-made UNA-620C modules, Rack2 with Chinese-made UNA-620C modules, and Rack3 with Japanese-made WTR-620C modules (WTR-60C is equivalent to the UNA-620C module). Figure 2 shows a photo of the membrane installation, while in Table 1, the characteristics of the MICROZA UNA-620C module are presented.

The membrane material used in the analyzed plant was very popular and widely applied by water utilities. Polyvinylidene fluoride (PVDF) is a benchmark material for water treatment membranes (MF and UF) due to its outstanding mechanical strength and high chemical tolerance against harsh cleaning agents like oxidants. The widespread use of these membranes is confirmed by the literature [20,21].

### 2.2. Membrane Cleaning Procedures

The membrane operating cycle includes:

- Main filtration (30 min);
- Air scrubbing (45 s)—cleaning the capillary surfaces with air bubbles (AS);
- Backwash (45 s)—rinsing the capillaries with permeate (BW). According to the system supplier, backflush flux ranged from 80 to 120 L/m^2^·h, while the maximum allowable backwash pressure amounted to 2.5 bar. The backwash parameters were adjusted by the operator depending on the raw water quality and the resulting backwashing efficiency.

Additionally, to remove organic and inorganic foulants from the membrane surface and pores that were not eliminated during the air/water cleaning, the membranes undergo chemical cleaning using acidic and basic solutions (standard chemical cleaning). Moreover, in accordance with the membrane manufacturer’s recommendations, once a quarter, in-depth chemical cleaning of the membranes is carried out in accordance with the CIP (clean-in-place) procedure. The chemical cleaning parameters are listed in Table 2.

Each time after chemical cleaning, the membranes and membrane modules are rinsed with water. It is estimated that approximately 120–130 m^3^/d of water is consumed for membrane rinsing.

### 2.3. Membrane Resistance Calculation

The resistance of membranes was calculated according to Darcy’s law:

$$J=\frac{∆P}{\mu \cdot R}$$

and the hydraulic permeability of the membrane is defined as

$$L_{p}=\frac{J}{∆P}\Rightarrow J=L_{p}\cdot ∆P$$

Summarized from those equations, the resistance was calculated as:

$$R=\frac{1}{µ\cdot L}$$

R—membrane resistance (m^−1^);
L—permeability at temperature T (m^3^·m^−2^·h^−1^·bar^−1^);
µ—dynamic viscosity of water at temperature T (Pa·s).

## 3. Results

### 3.1. Raw Water Characteristics

Raw water abstracted from the San River is characterised by stable physical, chemical and microbiological composition over many years and variability in composition typical for surface waters. The water was found to contain significant amounts of indicator microorganisms. A significant deterioration in water quality, particularly in terms of turbidity and colour, is observed in the spring season and in other periods following heavy rainfall in the San River basin. As shown by the analysis of literature data, the quality of raw water in the analysed period is similar to that observed in previous years [22]. The variability of selected physical, chemical, and microbiological parameters of raw water is presented in Table 3.

### 3.2. Analysis of Water Quality Parameters Across Treatment Stages

As a pretreatment step for microfiltration, surface water is subjected to batch coagulation followed by sedimentation and rapid filtration, effectively reducing the overall pollutant load and microbial count (Table 4 and Table 5). During the study, coagulation and sedimentation achieved an average reduction of 91.5% for turbidity and 50.1% for colour, significantly improving water quality towards potable standards. Despite significant microbial reduction—averaging 97.1% for coliforms, 99.2% for *E. coli*, 84.9% for *Clostridium perfringens*, and 90.2% for *Faecal streptococci*—periodic residual concentrations in coagulated water remained notably high. During periods of deteriorated raw water quality, elevated coagulant dosages are required (up to 9.8 g Al/m^3^), leading to high residual coagulant levels (up to 825 µg Al/L) in the effluent. The purpose of rapid filters is to remove suspended solids, which results in a further reduction in water turbidity by an average of 98.1% (compared to raw water). Regarding microorganisms, periodic increases in microbial counts were observed in the filtrate compared to the settled water. This suggests the release of biomass from the biological film formed on the filter bed. These findings highlight the need for further treatment to ensure compliance with microbiological water quality standards. One such application is the microfiltration process utilized at the Jarosław WTP.

### 3.3. Separation and Transport Properties of Microfiltration Membranes

**Figure 3:**
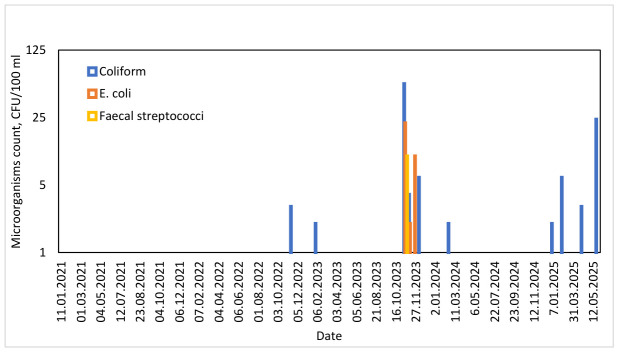
The content of microorganisms in water after microfiltration.

As illustrated in Figure 3, microbiological analyses of the permeate demonstrated complete microbial removal in the vast majority of samples. Only a few water samples—mostly from November 2023—tested positive for microorganisms, primarily coliforms. Identifying the exact cause of this presence after microfiltration is challenging, as no microbiological analysis was performed on the sand filter effluent during that period. These trace amounts of bacteria in the permeate do not constitute a health hazard (particularly since the water is also chemically disinfected); they likely result from variable flow rates in the microfiltration and UV treatment stages. Furthermore, the analysis reflects bacterial levels within a mixture of both streams.

The microfiltration membranes utilized at the Jarosław water treatment plant (WTP) feature an average pore diameter of 0.1 µm, which implies a rejection efficiency exceeding 90% for contaminants of at least 0.1 µm in size. Given the most common manufacturing process for polymer membranes (phase inversion), their structure inevitably contains pores both smaller than the nominal diameter—enabling the retention of particles smaller than specified—and larger than the nominal diameter, which allows for the passage of particles that should theoretically be rejected. Furthermore, bacterial cells are capable of deformation, which may facilitate their transport through the membrane [23]. According to literature data, the dimensions of *E. coli* are 2 µm/0.25–1 µm [24], *Clostridium perfringens* are 4–8 µm/0.8–1.5 µm [25], while *Faecal streptococci* cells are spherical with a diameter of 0.5–1 µm. Therefore, the penetration of individual cells through a membrane with a nominal pore diameter of 0.1 µm is plausible.

It can therefore be concluded that despite their long-term operation, the MF membranes generally maintain high microbial separation efficiency. However, the increased frequency of coliform detection in 2025 may suggest a gradual decline in their rejection properties. This may be a consequence of pore enlargement caused by membrane degradation as a result of long-term use of NaOCl for membrane cleaning [26]. Membrane ageing may also alter surface properties, thereby promoting biofilm formation and growth. As a consequence, the efficiency of microorganism retention may gradually decrease [10].

**Figure 4:**
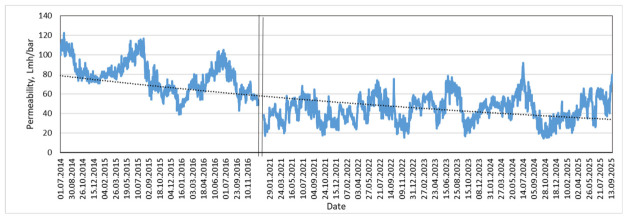
Changes in permeability during membrane operation (dotted line–trend line).

**Figure 5:**
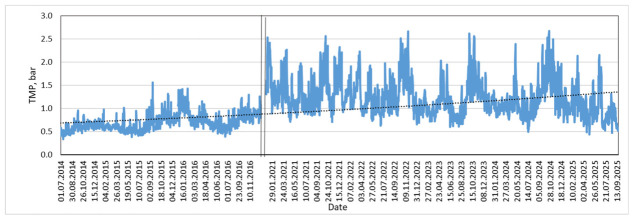
TMP variations during long-term membrane operation (dotted line–trend line).

The fundamental issue identified during long-term membrane operation is the deterioration of transport properties, resulting in a decline in permeability and an increase in transmembrane pressure. As illustrated in Figure 4 and Figure 5, this phenomenon is also evident in the membranes operated at the Jarosław WTP. While in the initial period (2014–2016) this was primarily associated with seasonal water temperature fluctuations affecting dynamic viscosity, in the years 2021–2025, membrane aging also contributed to the degradation of transport properties. Both the increase in water dynamic viscosity and intensified membrane fouling lead to higher membrane resistance. As shown in Table 6, the resistance to water flow through the operated membranes has increased nearly threefold over the period from 2014 to 2025.

A review of the literature suggests [10,23] that the gradual increase in the resistance of PVDF membranes during long-term operation may result from enhanced hydrophobicity caused by the removal of hydrophilicity-enhancing additives from the membrane material. During long-term operation, the membranes may also experience capillary damage within the module, which can further contribute to a decline in membrane performance [27].

**Figure 6:**
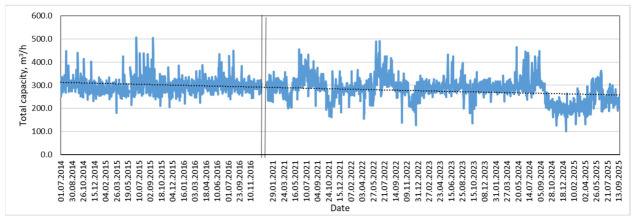
Total membrane system capacity (dotted line–trend line).

As illustrated in Figure 6, compensating for increasing flow resistance by raising the TMP allowed a nearly constant membrane plant capacity (approx. 300 m^3^/h) to be maintained for many years, meeting the water demand of the supplied area. Periodically, during summer seasons, the plant’s capacity approached 500 m^3^/h, which, among other factors, was the result of the previously described changes in water properties. As shown in Figure 5, maintaining the expected system output necessitated the use of an ever-increasing driving force (TMP), which more and more frequently approached the limit value specified by the manufacturer (3.0 bar).

In accordance with common practice and the manufacturer’s recommendations, the membranes operated at the Jarosław WTP are systematically subjected to chemical cleaning, which aims to remove organic and inorganic substances responsible for fouling from the membrane surface and pores. The parameters applied for chemical cleaning are summarized in Table 2. Acids, alkalis, and hypochlorite are the chemicals used for membrane cleaning. According to literature data [28], PVDF membranes are relatively stable under acidic conditions but can degrade in strongly alkaline environments. Strong oxidants, such as sodium hypochlorite, may also attack the PVDF structure. Furthermore, hydrophilic modifiers such as polyvinyl pyrrolidone (PVP) or polyethylene glycol (PEG), used for pore creation and surface functionalization, are susceptible to leaching during chemical cleaning [29]. This depletion of additives can subsequently accelerate the aging process of the membrane.

**Figure 7:**
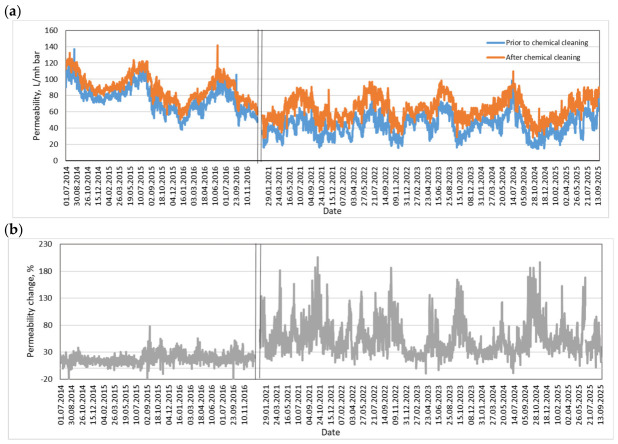
The influence of chemical cleaning on membrane transport properties: (**a**) permeability, (**b**) the percentage of permeability recovery.

As illustrated in Figure 7, chemical cleaning of the membranes contributes to an increase in their permeability, indicating the effective removal of contaminants deposited on the membrane surface. In the period 2014–2016, the increase in membrane permeability resulting from chemical cleaning was relatively small, averaging approximately 19%. In the years 2021–2025, significantly higher increases in permeability were observed after chemical cleaning; however, the fluctuations in these changes were much greater than in the earlier period. This may confirm the cited information regarding changes in the properties of the membrane-forming polymer, resulting in a higher susceptibility of the membranes to fouling.

Despite the application of systematic backwashing and chemical cleaning during membrane operation, the membrane permeability is significantly lower than in the initial period of operation (at the turn of 2024/2025, a decrease of 86.4%), which indicates irreversible fouling.

Changes in the transport properties of the membranes in Jarosław are the result of the simultaneous occurrence of irreversible fouling and membrane aging, which proceed concurrently and affect the efficiency of water treatment. Irreversible fouling and membrane aging represent two distinct phenomena affecting PVDF performance. Irreversible fouling is mainly associated with the accumulation of organic, inorganic, and biological matter on the membrane surface and within pores, leading to partial flux decline that can be only partly restored by chemical cleaning and is not accompanied by permanent changes in polymer structure. In contrast, membrane aging induced by long-term exposure to sodium hypochlorite is a chemical and irreversible process involving degradation of the polymer matrix and especially the loss of hydrophilic additives (e.g., PVP). This results in permanent changes in membrane morphology and surface chemistry, including pore structure alteration and increased surface roughness, as confirmed by FTIR and XPS analyses [30,31]. Thus, fouling is primarily a reversible operational phenomenon, whereas aging reflects long-term structural degradation of the membrane material.

A 70% reduction in permeability is commonly used by managers of membrane plants as a replacement trigger because it reflects a significant unjustifiable loss of membrane performance [32]. The cost of replacing 120 membrane modules will amount to approximately 480,000 euros [33].

## 4. Conclusions

1. Long-term (11-year) operation validates full-scale microfiltration (MF) as a highly reliable and robust barrier for surface water treatment, maintaining excellent separation efficiency—specifically regarding turbidity and microbiological removal—throughout its entire lifecycle.
2. The evolution of membrane transport properties reveals a severe long-term degradation mechanism, where progressive aging and irreversible fouling led to a near-threefold increase in membrane resistance and a corresponding 86% decline in permeability. This highlights the long-term limits of hydraulic performance under real-world operating conditions.
3. While chemical and physical cleaning protocols are vital for flux maintenance, their efficiency exhibits increased variability over time. This trend points to irreversible structural modifications within the PVDF matrix, driven by decade-long exposure to alkaline and oxidizing cleaning agents, which subsequently enhances the material’s susceptibility to fouling.
4. The observed permeability loss surpasses the conventional 70% threshold for membrane replacement, confirming that asset lifecycle planning for full-scale MF plants must transition from purely time-based schedules to aging-driven models. Incorporating these long-term aging dynamics is essential for optimizing both the technical management and economic viability of municipal water treatment infrastructure.

## Figures and Tables

**Table 1:** The characteristics of the membrane module.

Module Symbol	MICROZA UNA-620C
Mean pore diameter	0.1 µm
Membrane material	PVDF
Membrane surface	50 m^2^
Internal capillary diameter	0.7 mm
External capillary diameter	1.4 mm
Direction of flow through the membrane	outside/inside
Max transmembrane pressure	3.0 bar
Max temperature	40 °C
pH range	1–10
Module length	2160 mm
Module diameter	165 mm
Capacity	~4 m^3^/h

**Table 2:** Parameters of chemical cleaning of membranes.

	Alkaline Cleaning	Acidic Cleaning
Standard chemical cleaning		
frequency	every 16–24 h	every 7 d
cleaning solution	NaOH + NaOCl	citric acid
cleaning solution temperature	30 °C	22 °C
duration	50 min	50 min
CIP		
cleaning solution	NaOH + NaOCl	C_6_H_8_O_7_
cleaning solution temperature	30 °C	22 °C
duration	100 min	70 min

**Table 3:** Raw water composition.

Parameter	Unit	Mean	Min	Max	SD
Colour	mg Pt/L	20.9	5	50	6.5
Turbidity	NTU	17.1	2.2	382	31.2
pH			7.4	8.2	0.1
Temperature	°C	11.9	0.1	28	
Conductivity	µS/cm	436	293	801	96.1
Total hardness	mg CaCO_3_/L	210	137	364	36.2
Cl	mg Cl/L	12.9	6	21	3.0
NO_2_	mg NO_2_/L	0.099	<0.006	0.419	0.1
NO_3_	mg NO_3_/L	3.04	0.051	9.39	2.6
NH_4_	mg NH_4_/L	0.071	0.008	0.17	
Fe	µg Fe/L	315	20	1362	236.0
Mn	µg Mn/L	280	49	824	198.6
Permanganate index (COD-Mn)	mg O_2_/L	3.4	1	7	1.1
Coliform	CFU/100 mL	3904	80	98,000	13,072
*E. coli*	CFU/100 mL	404	0	3000	609
*Clostridium perfringens*	CFU/100 mL	250	6	2000	295
*Faecal streptococci*	CFU/100 mL	310	4	2900	449

**Table 4:** Water quality after coagulation and sedimentation.

Parameter	Unit	Mean	Min	Max	SD
Colour	mg Pt/L	10.4	5	45	4.0
Turbidity	NTU	1.4	0.28	10	1.1
pH			6.5	8	0.2
Aluminium	µg Al/L	222	25	825	107.7
Coliform	CFU/100 mL	87	6	280	104
*E. coli*	CFU/100 mL	26	0	220	53
*Clostridium perfringens*	CFU/100 mL	36	0	250	59
*Faecal streptococci*	CFU/100 mL	29	0	97	32

**Table 5:** Water quality after rapid sand filtration.

Parameter	Unit	Mean	Min	Max	SD
Turbidity	NTU	0.34	0.13	2.6	0.3
Coliform	CFU/100 mL	111	0	1000	86
*E. coli*	CFU/100 mL	155	0	380	24
*Clostridium perfringens*	CFU/100 mL	16.2	0	246	9
*Faecal streptococci*	CFU/100 mL	8.4	0	96	21

**Table 6:** Changes in membrane resistances (m^−1^).

Year	Membrane Resistance, m^−1^	
	Winter (1 °C)	Summer (21 °C)
2014	3.55 × 10^12^	2.77 × 10^12^
2016	4.27 × 10^12^	3.72 × 10^12^
2021	8.29 × 10^12^	7.42 × 10^12^
2025	9.95 × 10^12^	8.64 × 10^12^

## Data Availability

The raw data supporting the conclusions of this article will be made available by the authors on request.
